# Screening and Validation of Housekeeping Genes of the Root and Cotyledon of *Cunninghamia lanceolata* under Abiotic Stresses by Using Quantitative Real-Time PCR

**DOI:** 10.3390/ijms17081198

**Published:** 2016-07-28

**Authors:** Wenlong Bao, Yanli Qu, Xiaoyi Shan, Yinglang Wan

**Affiliations:** College of Biological Sciences and Biotechnology, Beijing Forestry University, 35 Qinghua East Road, Haidian District, Beijing 100083, China; baowenlong@bjfu.edu.cn (W.B.); quyanli@bjfu.edu.cn (Y.Q.)

**Keywords:** Chinese fir, housekeeping gene, NormFinder, BestKeeper, geNorm, RankAggreg

## Abstract

*Cunninghamia lanceolata* (Chinese fir) is a fast-growing and commercially important conifer of the Cupressaceae family. Due to the unavailability of complete genome sequences and relatively poor genetic background information of the Chinese fir, it is necessary to identify and analyze the expression levels of suitable housekeeping genes (HKGs) as internal reference for precise analysis. Based on the results of database analysis and transcriptome sequencing, we have chosen five candidate HKGs (*Actin*, *GAPDH*, *EF1a*, *18S rRNA,* and *UBQ*) with conservative sequences in the Chinese fir and related species for quantitative analysis. The expression levels of these HKGs in roots and cotyledons under five different abiotic stresses in different time intervals were measured by qRT-PCR. The data were statistically analyzed using the following algorithms: NormFinder, BestKeeper, and geNorm. Finally, RankAggreg was applied to merge the sequences generated from three programs and rank these according to consensus sequences. The expression levels of these HKGs showed variable stabilities under different abiotic stresses. Among these, *Actin* was the most stable internal control in root, and *GAPDH* was the most stable housekeeping gene in cotyledon. We have also described an experimental procedure for selecting HKGs based on the de novo sequencing database of other non-model plants.

## 1. Introduction

The conifers of division *Pinophyta* are considered to be of great ecological importance in most areas of the northern hemisphere. From the temperate zone to the northern tundra, conifers are predominant plants of temperate coniferous and boreal forests [1,2,3]. The conifers do not only have indispensable ecological roles in the Earth’s biosphere, but also have critical economic contribution to various countries. The Chinese fir (*Cunninghamia lanceolata* (Lamb.) Hook) is one of the most extensively cultured evergreen conifers in south China. It is native to China and also widely distributed in temperate regions in the north of Vietnam. Chinese fir is a fast-growing conifer that belongs to family Cupressaceae, and is a commercially important tree of the lumber, pulp, and paper industry. Biological studies have recently revealed the molecular mechanism underlying the control of the developmental processes and physiological responses of the Chinese fir [4,5].

As a perennial woody plant that differs from herbaceous plants, the Chinese fir can live for hundreds of years. During this long life cycle, it is subjected to various changes in its natural environment, including temperature, water, and soil nutrients. Early studies investigated the effects of growth conditions on its morphological characters and biochemical components [3,6]. Using advanced genetic approaches, researchers have recently focused on elucidating the molecular mechanisms underlying the regulation of physiological responses, including analyses of gene function [7,8], transcriptome [4,9], or small non-coding RNA and miRNA identification [5,10]. Although next-generation sequencing (NGS) technologies allow ultrahigh-throughput and highly accurate quantification of gene expression levels, the associated high cost of sequencing and post-verification on extensive amounts of nucleic acids (DNA or RNA) limit its widespread use [11,12,13]. In this context, quantitative real-time polymerase chain reaction (qRT-PCR) analysis is a conventional choice for post-verification of the transcriptome data and to facilitate in-depth expression studies of smaller sets of genes, including studies of alternative splicing, verification of microarray expression results, and molecular diagnostics [14,15,16,17].

qRT-PCR is a well-established technique for quantifying the expression levels of target genes [9,18]. Approaches for detection of the PCR products amount (amplicon) using qRT-PCR are classified into two categories: relative quantification based on housekeeping genes (HKGs) and absolute quantification achieved with DNA standards via calibration curve [19]. Relative quantification is one of the most straightforward and robust methods for accurately quantifying subtle changes in gene expression. To avoid biased results and erroneous interpretations, a critical component of relative quantification analysis is to normalize data by measuring in parallel the expression of HKGs from the same specimen [20,21,22,23]. HKGs that are constitutively expressed to maintain cellular function, often referred to as reference genes or internal controls, are expressed at constant levels in different tissues and organs of specimens under various biotic and abiotic circumstances. As a basic prerequisite for relative quantification, HKGs have been extensively used to normalize transcript data generated by qRT-PCR as well as microarrays [24,25]. NGS data mining and HKGs identification in model species have shown that those of internal controls exhibited not only species-specific expression, but also tissue-specific expression. Furthermore, their expression levels are also influenced by environmental factors (water, light, and temperature), as well as specific experimental conditions [19,24,26,27,28].

In general, HKGs such as 18S ribosomal RNA (*18SrRNA*), beta-actin (*β-actin*), elongation factor-1 alpha (*EF1a*), ubiquitin (*UBQ*), and glyceraldehyde-3-phosphate dehydrogenase (*GAPDH*) have been widely adopted for normalization [29,30]. However, it has been suggested that *UBQ10* and *G6PD* should be avoided for gene expression normalization in soybean [31]. Therefore, two or more HKGs are used to normalize relative quantified data generated from qRT-PCR, which are expressed at constant levels under various experimental conditions [25,28]. Furthermore, there is an increasing number of attempts for HKG identification, including that of *Carica papaya* [32], *Solanum tuberosum* [33], *Linum usitatissimum* [19], *Kosteletzkya virginica* [29], *Oryza sativa* [16], *Lycopersicon esculentum* [34], *Triticum aestivum* [35], *Eucalyptus robusta* [36], *Arabidopsis thaliana* [37], *Sesamum indicum* [38], and *Populus euphratica* [39].

Conifers have the most complex and largest genomes, as well as diverse and highly diverged repetitive sequences among all living organisms; and therefore, sequencing and assembly of their complete genomes have been described as highly challenging [40,41,42]. Thus, it is essential to provide reliable methods for determining the gene expression pattern of conifers. However, only one study has screened HKGs in the *Pinus pinaster* [43]. Among 10 candidate endogenous references, *adenosine kinase* and *UBQ* were determined to be acceptable options, based on the observed low level of variation in *C*_t_ values and M values in the three developmental stages of *Pinus pinaster*. In contrast, several genes showed variable levels of expression with various conditions. However, no validation of HKGs upon abiotic stresses in conifers has been reported, and information on these particular genes in stress-tolerant herbaceous plants is limited. Because abiotic stresses such as heat, cold, salinity, and drought cause considerable losses in plant biomass and retard plant growth, it is important to breed stress-resistant plants.

Chinese fir is one of the most important conifers; however, its genomic information and systematic studies on validating HKGs for qRT-PCR studies are largely unknown, thereby hindering in-depth investigations on the functions of its gene. Herein, based on the transcriptomic data (GenBank Accession Numbers SRR2087198 and SRR2087918) from our previous study, we selected five common HKGs and used them to conduct BLAST analysis to identify highly conserved sequences. We then evaluated the following genes, *Actin*, *GAPDH*, *EF1a*, *18S rRNA*, and *UBQ*, in the roots and cotyledons of Chinese fir seedlings under five different abiotic stresses, namely, cold, high temperature (HT), abscisic acid (ABA), polyethylene glycol (PEG), and sodium chloride (NaCl). The data generated from qRT-PCR were then further analyzed using comprehensive and accurate tools for statistical analysis, such as NormFinder [28], geNorm [44], BestKeeper [45], and RankAggreg [46], to determine the most appropriate one.

## 2. Results

### 2.1. Quality Control

**Figure 1:**
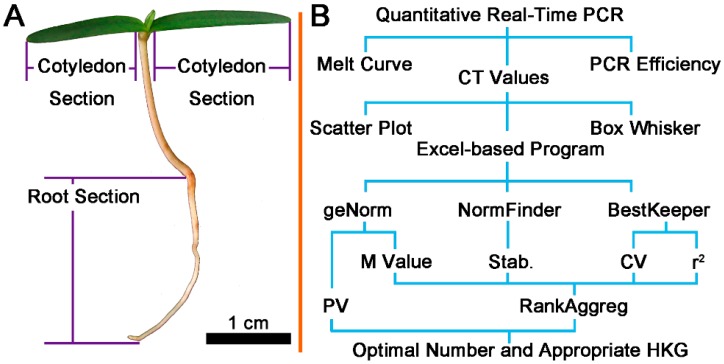
Sample sets and data analysis flow chart: (**A**) different section of Chinese fir used in experiment; and (**B**) data analysis flow chart. Cycle threshold (*C*_t_) values were calculated using different algorithms. Each candidate HKG has one efficiency value. The *C*_t_ data were checked for distribution by scatter plot and box whisker. The statistical results from three excel-based program were merged with RankAggreg. Abbreviations: Stab.: NormFinder stability value; *r*^2^: determination coefficient-regression from BestKeeper; CV: coefficient of variance from BestKeeper; M: classical stability value from geNorm; PV: pairwise variation from geNorm.

We selected the cotyledon and root section of Chinese fir, which were marked in Figure 1A and all data were analyzed according to the flow chart presented in Figure 1B.

RNA samples were evaluated by the following quality controls: (1) We used NanoDrop2000 spectrophotometer (Thermo, Waltham, MA, USA) to measure the OD_260_/OD_280_ and OD_260_/OD_230_ ratios. As measured, the OD_260_/OD_280_ ratio was between 1.8 and 2.0, and the OD_260_/OD_230_ ratio was >1.7; (2) 25S ribosomal RNA bands in the electrophoresis gel were about two-fold sharper than the 18S ribosomal RNA bands (Figure S1); (3) Amplicons generated from RT-PCR showed the predicted monomer size and did not form any primer-dimers (Figure S2A). The PCR products of these candidate HKGs were then subcloned into a T-vector for sequencing. In addition, primer specificity was further validated using melting curve analysis (Figure S2B–F) and sequencing consequences. The PCR efficiencies of these five candidate HKGs varied from 1.92 to 2.08, of which *EF1a* had the lowest amplification efficiency and *GAPDH* showed the highest efficiency, followed by *Actin*, *UBQ*, and *18S*. The transcriptome ID, *Arabidopsis* orthologous locus, primer sequences, amplicon length, and PCR efficiencies of the five candidate HKGs in Chinese fir are listed in Table S1.

### 2.2. Transcriptional Patterns

**Figure 2:**
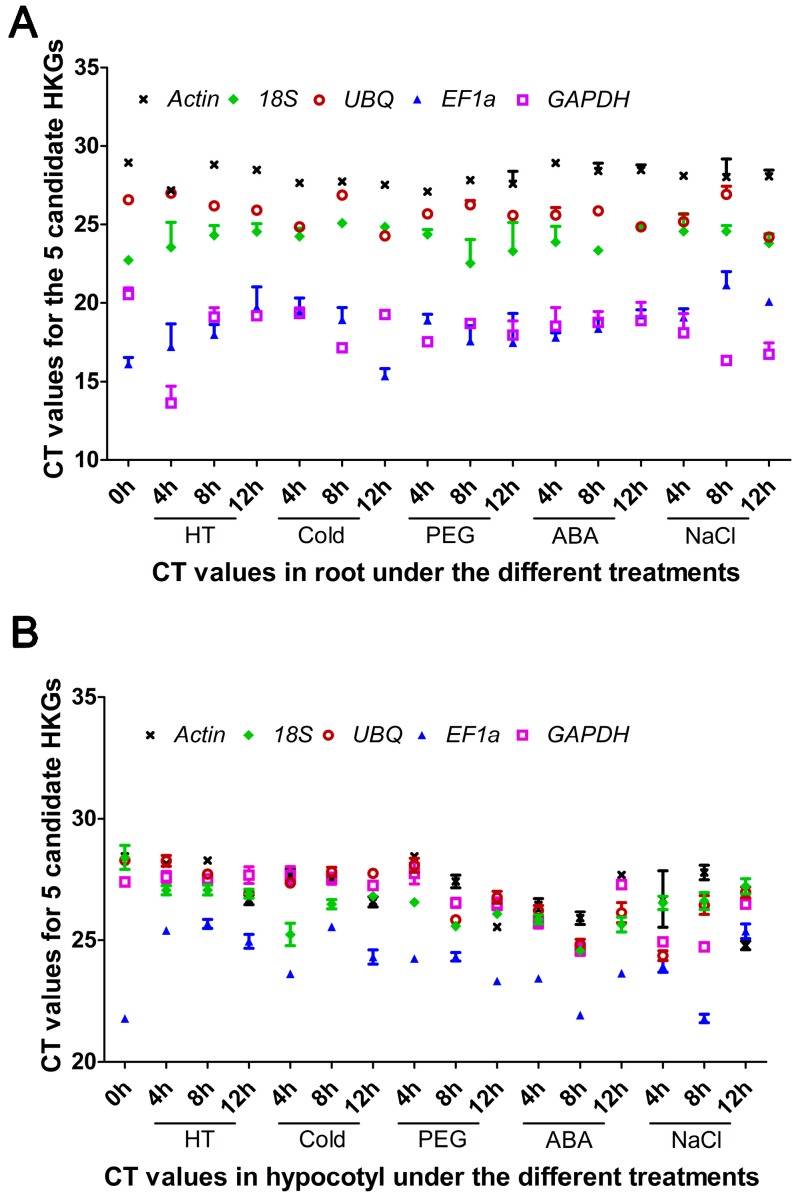
Transcription abundance levels of HKGs tested in Chinese fir root (**A**) and cotyledon (**B**), shown as *C*_t_ mean value ± Standard Deviation (SD). Abbreviations: HT: High Temperature treatment; Cold: Cold treatment; ABA: Abscisic Acid treatment; NaCl: Sodium Chloride treatment; PEG: Polyethylene Glycol treatment.

**Figure 3:**
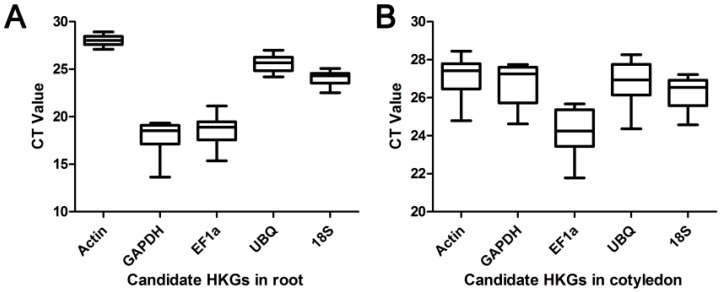
*C*_t_ variation among experimental sets of Chinese fir root (**A**) and cotyledon (**B**), shown as Box-whisker plot. The median is presented as the line across the box. The upper and lower edges bordered in each box are interquartile range, which indicate the 25th and 75th percentiles.

The raw cycle threshold [47] values generated from qRT-PCR were analyzed and results showed variation among candidate HKGs in all tested samples. In roots, the average *C*_t_ values of the five HKGs that were subjected to different treatments ranged from 13.63 to 28.92 (Figure 2A). The gene encoding *GAPDH* had the highest expression level, which reached its cycle threshold after only 13.63 amplification cycles, whereas the *C*_t_ value of *Actin* with 28.92 exhibited the lowest transcript abundance. Unlike the root, the *C*_t_ values of these genes in cotyledon samples were between 21.78 (*EF1a*) and 28.45 (*Actin*) (Figure 2B). In all tested samples, *Actin* showed the highest *C*_t_ values in both roots and cotyledons. Each HKG displayed different levels of expression in various tissues under the same treatment. *GAPDH* and *EF1a* showed a higher expression level in root samples than in cotyledon samples. Furthermore, except for the *C*_t_ values of *GAPDH* and *EF1a* in roots, the rest of the *C*_t_ values of HKGs were within the range of 22–29. The data distribution is shown as a box-whisker in Figure 3; in roots, the *Actin* (SD = ±0.53) had the least variable transcript abundance, which is reflected by its low SD values. On the contrary, *GAPDH* showed the most variable transcript abundance (SD = ±1.47) (Figure 3A). In cotyledons, the greatest expression variation was observed in *EF1a* (SD = ±1.16), whereas that of *18S* (SD = ±0.75) was the most stable (Figure 3B). These results indicated that no single candidate HKG had a constant expression level in different Chinese fir samples under five abiotic stresses, and thus it is of great importance to screen the best HKG(s) for gene expression normalization under certain experimental conditions in Chinese fir.

### 2.3. Statistical Analysis

#### 2.3.1. NormFinder

NormFinder was used to evaluate the expression stability of the candidate HKGs on the experimental samples. Raw *C*_t_ values were first log-transformed and used as input for the NormFinder. This algorithm takes into account the intra- and inter-group variations for normalization factors (NFs), which requires input data from a minimum of three candidate HKGs and a minimum of two tested samples per group. The calculated results of this software were not influenced by random co-regulated genes. The best candidate HKG displayed higher stability values that were close to zero. Table 1 and Table 2 present the stability values of tissue-specific and condition-dependent HKGs. With cold treatment, the best HKG in root was *Actin* (stability value: 0.009), whereas in the cotyledon it was *UBQ* (0.008). With HT treatment, in root, the best HKGs were *Actin* and *18S* (0.007), and these two genes were also identified as the most stably expressed genes during NaCl treatment. In the cotyledon, *EF1a* (0.006) was the optimal HKG during HT stress, and *GAPDH* and *18S* (0.012) were the most stable HKGs during NaCl stress. The *GAPDH* showed to be the optimal candidate in roots under the ABA treatment, which was also evaluated as the best candidate in cotyledons under the PEG treatment. *UBQ* (0.006) was the best HKG in the root during PEG stress, and *EF1a* (0.008) was the most stable gene in the cotyledon during ABA stress. Among all treatments, *Actin* (0.017) was the most stable candidate in root samples and *GAPDH* (0.095) was the least stable. Furthermore, *GAPDH* (0.021) showed the least variation in cotyledon samples and Actin had the highest (0.043).

#### 2.3.2. BestKeeper

BestKeeper was applied to rank the stability values by calculating the coefficient of variance (CV). The candidate HKGs are considered to be the most stably expressed genes when they present the lowest CV. The BestKeeper index reflects another function of the BestKeeper program, which is conducted using the coefficient of determination (*r*^2^). The *r*^2^ is calculated to identify a credible NF, but not to estimate the goodness of each HKG independently. Therefore, the closer *r*^2^ is to 1, the better. In the present study, we applied both CV and *r*^2^ to rank the stability values of five candidate HKGs. According to CV values generated from BestKeeper, *Actin* was identified as the best HKG for cold-, PEG-, and NaCl-treated root, and *UBQ* and *GAPDH* showed the greatest stability in HT- and ABA-treated root, respectively (Table 3). As shown in Table 4, except for *18S* in NaCl-treated cotyledon, none of the candidate genes in all of the sample sets tested were ranked as the best one by both CV and *r*^2^. For example, for the root under PEG stress, only considering the CV, *Actin* showed the most stable expression pattern, but based on *r*^2^ ranking, *EF1a* was the best (Table 3). In the cotyledon, the best HKG in PEG, ABA, and NaCl treatments was *18S*, and *GAPDH* was the most stably expressed gene in both HT and cold treatment (Table 4). Altogether, based on CV values, *Actin* was identified as the best HKG in all tested root samples (Table 3), whereas *18S* was the most stably expressed gene in all tested cotyledon samples (Table 4). However, as ranked by *r*^2^, *EF1a* was the best HKG for cold-, PEG-, and ABA-treated roots, and *GAPDH* and *UBQ* showed the greatest stability in HT- and NaCl-treated roots, respectively (Table 3). In cold- and ABA-treated cotyledons, *EF1a* was the best HKG, whereas the genes *Actin*, *GAPDH*, and *18S* showed the greatest stability in HT-, PEG- and NaCl-treated cotyledons, respectively (Table 4). In conclusion, according to *r*^2^, *EF1a* was the most stable HKG in all root samples, and *GAPDH* exhibited the lowest variability in all cotyledon sample sets (See the specific data of CV and *r*^2^ in Tables S2 and S3).

#### 2.3.3. geNorm

**Figure 4:**
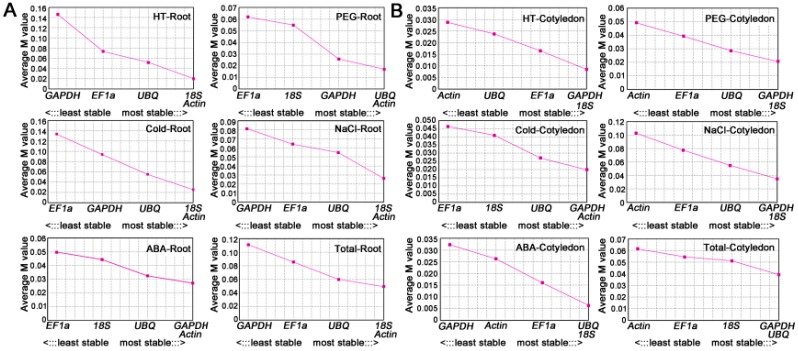
*M* values (average expression stability) and ranking of the candidate HKGs in Chinese fir root (**A**) and cotyledon (**B**) as calculated by geNorm. A lower *M* value indicates more stable expression. HT: High Temperature treatment; Cold: Cold treatment; ABA: Abscisic Acid treatment; NaCl: Sodium Chloride treatment; PEG: Polyethylene Glycol treatment.

geNorm (Version 3.5, Ghent, Flanders, Belgium) was used to identify the expression stability of candidate HKGs based on the *M* value, which is defined as the average variation of a certain gene with regard to all other candidate HKGs. The gene with the lowest *M* value is considered to have the more stable expression, or conversely. According to this principle, the geNorm program will stepwisely exclude the least stable gene and be repeated until only two genes remain. The *M* values of the candidate HKGs are presented in Figure 4. In the ABA treatment, the *Actin* and *GAPDH* genes were ranked lowest in root samples, with an *M* value of 0.0271 (Figure 4A), whereas the *UBQ* and *18S* genes were most stably expressed in cotyledon samples, with an *M* value of 0.0063 (Figure 4B). For cold/HT/NaCl-treated root samples, the most stable genes were *Actin* and *18S* with *M* values of 0.0252, 0.02, and 0.0263, respectively (Figure 4A). The same most stably expressed HKGs were *GAPDH* and *18S* in HT/NaCl/PEG-treated cotyledon samples, with *M* values of 0.085, 0.0351, and 0.0207, respectively (Figure 4B). The *Actin* and *UBQ* genes performed best in PEG-treated root samples, with an *M* value of 0.0169 (Figure 4A), whereas the *Actin* and *GAPDH* genes were ranked lowest, with an *M* value of 0.0199, in cold-treated cotyledon samples (Figure 4B). For all tested sample sets, the genes encoding *Actin* and *18S*, with an *M* value of 0.0494, showed the most stable expression HKGs in roots (Figure 4A). Furthermore, *GAPDH* and *UBQ* were ranked lowest in cotyledon under different abiotic stresses, with an *M* value of 0.0392 (Figure 4B). In most tested samples, the results of geNorm were in high agreement with the results of NormFinder, although with slight variations in the ranking sequence of genes. In addition, regardless of the variations in ranking, these programs identified the same most stably expressed gene in all experimental samples.

**Figure 5:**
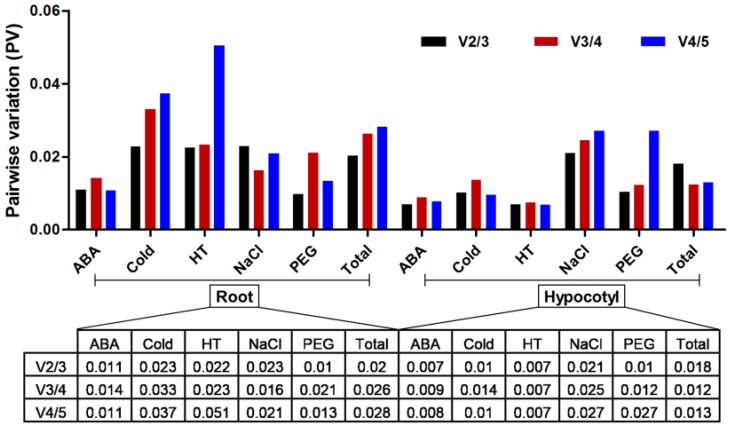
Pairwise variation (PV) to identify the optimal number of HKGs for precise normalization. The PV (V_n_/V_n+1_) was analyzed between the NFs (Normalization Factors) NF_n_ and NF_n+1_ using geNorm program. Abbreviations: HT: High Temperature treatment; Cold: Cold treatment; ABA: Abscisic Acid treatment; NaCl: Sodium Chloride treatment; PEG: Polyethylene Glycol treatment.

To determine the optimal number of HKGs for accurate normalization in different sample sets, we applied the geNorm program to further calculate the pairwise variation (PV) of two sequential normalization factors (NFs) (V_n_/V_n+1_) as standard deviation (SD) of the log-transformed NF_n_/NF_n+1_ ratios [44]. The NF is calculated according to the geometric mean of the number of candidate HKGs and the stepwise inclusion of other genes that were ranked based on its expression stability. Once the PV value of a candidate HKG is higher than the default cutoff value of 0.15, the additional HKG is considered to have a significant effect on normalization. Figure 5 shows the results of PV analysis, suggesting that normalization requires the adoption of only two HKGs in all tested samples because the V_2/3_ value of all tested samples was <0.15, the default cutoff value.

#### 2.3.4. RankAggreg

**Figure 6:**
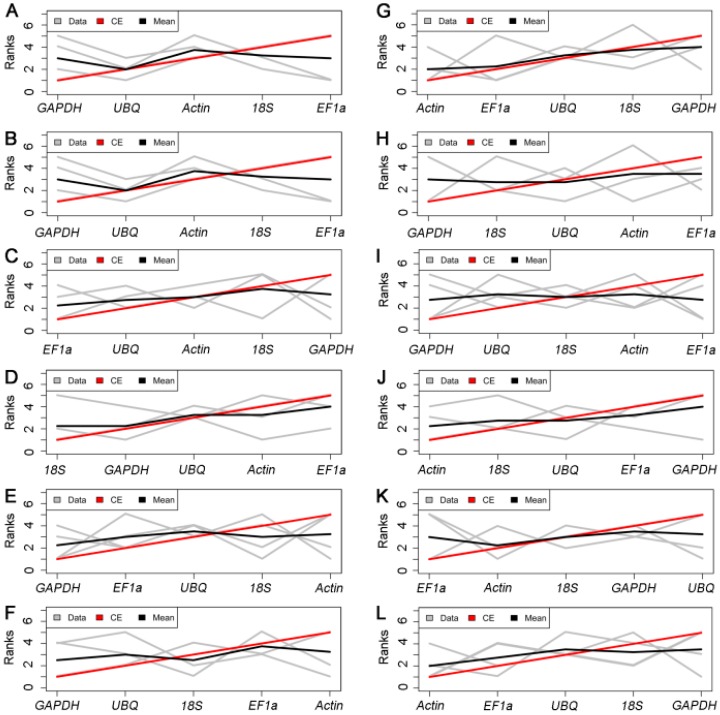
Rank aggregation of gene lists using the Monte Carlo algorithm. Visual representation of rank aggregation using Monte Carlo algorithm with the Spearman foot rule distances: (**A**–**F**) different treatments for cotyledon ((**A**) cold-treated; (**B**) HT-treated; (**C**) ABA-treated; (**D**) NaCl-treated; (**E**) PEG-treated; and (**F**) total); and (**G**–**L**) different treatments for roots ((**G**) cold-treated; (**H**) HT-treated; (**I**) ABA-treated; (**J**) NaCl-treated; (**K**) PEG-treated; and (**L**) total). Different lines in the plot represent the following: gray lines, stability measurement; black lines, rank position; and red lines, model computed by the Monte Carlo algorithm. Abbreviations: HT: High Temperature treatment; Cold: Cold treatment; ABA: Abscisic Acid treatment; NaCl: Sodium Chloride treatment; PEG: Polyethylene Glycol treatment.

To determine the suitability of HKGs for normalization of target gene transcript abundance, the ranking patterns of the three Excel-based programs were compared. To avoid discrepancies, ranking in candidate HKGs due to the use of different algorithms (Tables S4 and S5), we applied the RankAggreg approach to generate a consensus list. We combined the four outputs (M values from geNorm, stability values from NormFinder, and CV and *r*^2^ values from BestKeeper) for comprehensive ranking analysis. The merged data revealed that the best HKGs for normalization were: *GAPDH* for cold- (Figure 6A), HT- (Figure 6B), and PEG-treated cotyledon (Figure 6E) and HT- (Figure 6H), and ABA-treated root (Figure 6I); *EF1a* for ABA-treated cotyledon (Figure 6C) and PEG-treated root (Figure 6K); *Actin* for cold- (Figure 6G) and NaCl-treated root (Figure 6J); and *18S* for NaCl-treated cotyledon (Figure 6D). Considering all of the treatments, *GAPDH* was the best HKG for the cotyledon (Figure 6F), and *Actin* was the most stable HKG for the root (Figure 6L). On the basis of the HKGs number to use suggested by geNorm and the ranking list indicated by RankAggreg, the best combination of candidate HKGs in each treatment is presented in Table 5.

## 3. Discussion

Chinese fir is one of the most commercially important trees in China. Its lack of complete genome information has hindered the in-depth functional analysis of its genes. To provide a powerful tool for the quantitative analysis of this long-living tree, we evaluated five candidate HKGs in the cotyledon and root of the Chinese fir under various abiotic stresses. Based on the transcriptomic data of our previous study, we selected five candidate HKGs and BLASTed these with the genome sequences of other coniferous trees, including *Picea abies* and *Pinus taeda* in NCBI. We then obtained highly conserved sequences and used these in designing primers and cloning gene segments. This strategy not only provided reliable gene segments for cloning HKGs in Chinese fir, but also served as a foundation for cloning of these genes in other species.

As a sessile organism, the Chinese fir has to tolerate various abiotic and biotic changes in the environment such as light intensity, temperature, water, and nutrients, which are the most crucial signals that influence the metabolism, gene expression patterns, morphology, and development of plants [48,49]. Previous reports have shown that plants are equipped with an induction system for various stresses with miscellaneous sensors, including membrane proteins, second messengers, and transcription factors [50,51]. Under the stress stimuli, these sensors translate the signals to a downstream response to resist tolerance, which is then followed by unpredictable changes in transcript abundance of essential genes. In the present study, *Actin* in the root showed minimal variation under the five stresses, and *GAPDH* in the cotyledon was the most stably expressed gene in these abiotic treatments.

Generally, housekeeping genes such as *Actin*, *GAPDH*, *UBQ*, *18S*, and *EF1a*, which are used as endogenous reference genes in *Arabidopsis*, are also found to shift in some experimental sets [52]. Therefore, the identification of the HKGs in specific experimental conditions is necessary. To avoid erroneous expression estimates, we adopted three well-established statistical algorithms in our analysis, which are Excel-extended macro programs, Normfinder, Bestkeeper, and geNorm. In these three programs, NormFinder and BestKeeper test raw *C*_t_ values, whereas geNorm performs relative quantification, and the results thus identify discrepancies in PCR efficiency that may influence the validation of stability. We eliminated the influence of bias by using the two different statistical indices (*C*_t_-based CV and RQ-based *r*^2^) generated from BestKeeper, and combined the evaluation statistics. As identified previously from *Oryza sativa* [53], *Arabidopsis thaliana* [37], one of the most common HKGs, *UBQ*, was screened as the best HKG for PEG-treated root and cold-treated cotyledons. Another common HKG, *EF1a*, was validated as one of the best choices for *Brachiaria brizantha* [54]. In previous studies, scientists have applied and identified HKGs as internal control genes in different tissues of Chinese fir, including leaves, shoot apical meristems, stems, barks, xylems and vascular cambiums [4,5,55,56]. However, identification of HKGs in Chinese fir seedlings upon different abiotic stresses has not been reported. Our study showed that the *EF1a* was the best candidate as HKG for ABA-treated cotyledons (Figure 6C) and PEG-treated roots (Figure 6K). We then may infer that the HKGs identified in Chinese fir essentially correspond to the species mentioned above. Meanwhile, some HKGs suitable for qRT-PCR normalization were also identified in Chinese fir such as *Actin* for cold- (Figure 6G) and NaCl-treated roots (Figure 6J) and *18S* for NaCl-treated cotyledon (Figure 6D).

To avoid the biased results of different excel-based programs, we further used RankAggreg to merge the datasets. When considering all the treatments, the comprehensive results recommend that *GAPDH* is the best HKG for the cotyledon (Figure 6F) and *Actin* is the most stable HKG for the root (Figure 6L). According to the number of HKGs to use as calculated by geNorm and the consensus list suggested by RankAggreg, the best combination of candidate HKGs varied with treatment and tissues. For example, *GAPDH* and *UBQ* were the best HKGs for the cold-treated cotyledon, but *Actin* and *EF1a* were the best choice for the cold-treated root (Table 5). Taken together, these results indicate that HKGs show similar expression profiles in different tissues because these are involved in basic intracellular functions. However, in different species, some HKGs exhibited distinct expression patterns because each species has its own gene expression regulatory network. Therefore, it is necessary to systematically screen HKGs prior to its adoption in qRT-PCR normalization for specific organisms, even for different tissues, and developmental stages of identical species in certain experimental conditions.

To ensure accurate normalization, several authors have recommended the adoption of multiple HKGs in the analysis of gene expression [20,57,58,59]. For the Chinese fir abiotic stress study, we mainly focused on the impact of five abiotic treatments on the transcript stability of these HKGs. As expected, these candidate HKGs varied across different sample sets. Therefore, as PV values of V_2/3_ across all tested samples were <0.15 (Figure 5), we advocated two HKGs to be used for accurate quantification. Our findings, based on the RankAggreg data merged from three adequate estimated programs, reveal that the *GAPDH* gene may be used for normalization in cotyledon samples, whereas the *Actin* gene is the best internal control gene in roots. These results showed that gene expression stability is highly tissue-specific and external condition-dependent.

## 4. Materials and Methods

### 4.1. Sampling

The seeds of Chinese fir were obtained from Fujian Province, China. After 7 days of germination at 4 °C, the seeds were disinfected with 1% KMnO_4_ for 30 min and 0.2% HgCl_2_ for 10 min. Then, the seedlings were planted in sterile glass bottles under controlled conditions (28 °C/26 °C, 16-h-day/8-h-night cycle). There were 6 to 8 individuals in each glass bottle. After growth for 30 days, when the seedlings showed fully opened cotyledons and some true leaves, the plants were subjected to five different stress treatments: high temperature (37 °C), low temperature (4 °C), 100 μM abscisic acid (ABA), 250 mM sodium chloride (NaCl), and drought stress (20% polyethyleneglycol 4000, PEG4000). The specimens were sampled at 4, 8, 12 h after treatment. Moreover, for each sampling, the seedlings were separated to collect tissue samples of the roots and cotyledons. On the other hand, the seedlings, which were not subjected to stress treatment, were collected and marked as controls. All specimens were immediately frozen in liquid nitrogen and stored at −80 °C until analysis.

### 4.2. Total RNAs Extraction

Total RNAs were extracted from Chinese fir using a plant RNA purification reagent (Invitrogen, Carlsbad, CA, USA). First, frozen specimens were ground in liquid nitrogen to a fine powder with a pestle and a mortar. Second, the powder was completely dissolved in the plant purification reagent, and the mixture was centrifuged at 12,000× *g* at 4 °C for 2 min. This was then followed by the addition of chloroform to remove the DNA and isopropyl alcohol to recover the RNAs. Finally, the obtained RNA samples were examined by 2.5% agarose gel electrophoresis for 10 min.

### 4.3. cDNA Synthesis

Complementary DNA (cDNA) was synthesized using the Fast Quant RT Kit (TIANGEN, Beijing, China), following the manufacturer’s protocol. The reverse transcription system was based on 5 μg of total RNA, which generated approximately 20 μL of cDNA by using random primers. The resulting cDNAs were diluted to a ratio of 1:5 with nuclease-free water. Additionally, all of the cDNAs were stored at −20 °C until analysis.

### 4.4. Primer Design

As the complete genomes of Chinese fir were not published, we obtained the sequences of five genes from NCBI Sequence Read Archive SRA (GenBank Accession Numbers SRR2087198 and SRR2087918 for light- and dark-grown samples, respectively). The selected genes were used in BLAST analysis to identify highly conserved sequences, which were then used in designing primers with the Primer Premier 5. The length of the amplified fragments ranged from 150 to 200 bp. Finally, the control cDNAs was used as template to test the five pairs of primers by PCR to make sure that the primers were usable. The PCR products were extracted and cloned using a T vector and sequenced.

### 4.5. Quantitative Real-Time PCR (qRT-PCR)

qRT-PCR was implemented using the Kit (SuperRealPreMix Plus with SYBR Green from TIANGEN, Beijing, China) on a Real-Time PCR Detection System CFX96 (Bio-Rad, Hercules, CA USA). In addition, all cDNA templates used in the experiment were of the same concentration. Twenty microliters reaction systems were preparing using with following: 1 μL of the cDNA template, 7.4 μL of water, 10 μL of 2× SuperRealPreMix Plus, 0.4 μL of 50× ROX Reference Dye, and 0.6 μL of the forward and reverse primers. The PCR program involved a two-step process that was run for 40 cycles: 95 °C for 10 min, then denaturation at 95 °C for 10 s, annealing at 60 °C for 32 s, and extension at 72 °C for 10 s. Each reaction had four replicates. Melting curve data were gathered from 65 °C to 95 °C in 0.5 °C increments. The standard curve of each primer pair was established with serial dilutions of cDNA ((1/5)^0^, (1/5)^1^, (1/5)^2^, (1/5)^3^, (1/5)^4^ and (1/5)^5^). The amplification efficiency (E) of qRT-PCR was determined according to the equation: *E* = 10^−1/K^, where *K* represents the slope of the standard curve.

### 4.6. Bioinformatics and Statistical Analysis of Data

Excel-based programs were used in the present study, including geNorm, NormFinder and Best-Keeper. The RankAggreg package of the R program was applied to combine the four outputs, “Stab”, “CV”, “*r*^2^”, and “*M*”, which were generated from three Excel-based programs as earlier described. Based on the order of ranking, we employed a Monte Carlo algorithm to calculate and rank using a line chart. Because geNorm generated the same *M* value in the chart for the two least variable genes, we identified these two genes’ ranking with the initial *M* value that was calculated as the normalization factor (NF) value.

## 5. Conclusions

In summary, this work is the first in-depth study that has attempted to identify the optimal HKGs for the relative quantification of transcript abundance in Chinese fir using various abiotic stresses by means of qRT-PCR technology. We have validated the expression stabilities of five candidate HKGs in root and cotyledon sample sets from Chinese fir under five abiotic treatments in different time intervals. As a consequence, we recommend *Actin* and *GAPDH* for all tested root and cotyledon samples respectively as superior internal controls for normalization of qRT-PCR. In addition, our results indicated that different appropriate HKGs or a combination of HKGs for normalization should be screened based on different external conditions. We have also provided reliable HKG sequences and a solid foundation for the screening of HKGs in quantitative RT-PCR studies of transcript abundance in Chinese fir.

## Figures and Tables

**Table 1:** Expression stability values and ranking of HKGs in Chinese fir root as calculated by the NormFinder.

Rank Position	Cold-Root		HT-Root		NaCl-Root		ABA-Root		PEG-Root		Total-Root	
	Gene	Stab.	Gene	Stab.	Gene	Stab.	Gene	Stab.	Gene	Stab.	Gene	Stab.
1	*Actin*	0.009	*Actin*	0.007	*Actin*	0.009	*GAPDH*	0.009	*UBQ*	0.006	*Actin*	0.017
2	*18S*	0.023	*18S*	0.007	*18S*	0.009	*Actin*	0.016	*Actin*	0.017	*18S*	0.020
3	*UBQ*	0.038	*EF1a*	0.026	*UBQ*	0.043	*18S*	0.027	*18S*	0.042	*UBQ*	0.047
4	*GAPDH*	0.097	*UBQ*	0.087	*EF1a*	0.056	*EF1a*	0.035	*GAPDH*	0.042	*EF1a*	0.074
5	*EF1a*	0.126	*GAPDH*	0.174	*GAPDH*	0.070	*UBQ*	0.036	*EF1a*	0.043	*GAPDH*	0.095
Best gene	*Actin*		*Actin* and *18S*		*Actin* and *18S*		*GAPDH*		*UBQ*		*Actin*	
	0.009		0.007		0.009		0.009		0.006		0.017	

Abbreviation: HT: High Temperature treatment; Cold: Cold treatment; ABA: Abscisic Acid treatment; NaCl: Sodium Chloride treatment; PEG: Polyethylene Glycol treatment; Stab.: NormFinder stability value.

**Table 2:** Expression stability values and ranking of HKGs in Chinese fir cotyledon as calculated by the NormFinder.

Rank Position	Cold-Cotyledon		HT-Cotyledon		NaCl-Cotyledon		ABA-Cotyledon		PEG-Cotyledon		Total-Cotyledon	
	Gene	Stab.	Gene	Stab.	Gene	Stab.	Gene	Stab.	Gene	Stab.	Gene	Stab.
1	*UBQ*	0.008	*EF1a*	0.006	*GAPDH*	0.012	*EF1a*	0.008	*GAPDH*	0.007	*GAPDH*	0.021
2	*GAPDH*	0.016	*18S*	0.010	*18S*	0.012	*UBQ*	0.012	*18S*	0.019	*UBQ*	0.024
3	*Actin*	0.030	*UBQ*	0.013	*UBQ*	0.044	*Actin*	0.016	*EF1a*	0.024	*18S*	0.029
4	*18S*	0.030	*GAPDH*	0.019	*EF1a*	0.078	*18S*	0.017	*UBQ*	0.031	*EF1a*	0.035
5	*EF1a*	0.031	*Actin*	0.023	*Actin*	0.092	*GAPDH*	0.026	*Actin*	0.040	*Actin*	0.043
Best combination	*UBQ*		*EF1a*		*GAPDH* and *18S*		*EF1a*		*GAPDH*		*GAPDH*	
	0.008		0.006		0.012		0.008		0.007		0.021	

Abbreviation: HT: High Temperature treatment; Cold: Cold treatment; ABA: Abscisic Acid treatment; NaCl: Sodium Chloride treatment; PEG: Polyethylene Glycol treatment; Stab.: NormFinder stability value.

**Table 3:** Expression stability values and ranking of HKGs in Chinese fir root as calculated by BestKeeper.

Rank Position	HT-Root		Cold-Root		PEG-Root		ABA-Root		NaCl-Root		Total-Root	
	CV	*r*^2^	CV	*r*^2^	CV	*r*^2^	CV	*r*^2^	CV	*r*^2^	CV	*r*^2^
1	*UBQ*	*EF1a*	*Actin*	*GAPDH*	*Actin*	*UBQ*	*GAPDH*	*Actin*	*Actin*	*GAPDH*	*Actin*	*UBQ*
2	*18S*	*Actin*	*18S*	*18S*	*UBQ*	*GAPDH*	*Actin*	*GAPDH*	*18S*	*EF1a*	*18S*	*18S*
3	*Actin*	*UBQ*	*UBQ*	*UBQ*	*GAPDH*	*18S*	*UBQ*	*18S*	*EF1a*	*Actin*	*UBQ*	*GAPDH*
4	*EF1a*	*18S*	*GAPDH*	*Actin*	*18S*	*Actin*	*18S*	*UBQ*	*UBQ*	*18S*	*EF1a*	*Actin*
5	*GAPDH*	*GAPDH*	*EF1a*	*EF1a*	*EF1a*	*EF1a*	*EF1a*	*EF1a*	*GAPDH*	*UBQ*	*GAPDH*	*EF1a*

Abbreviation: HT: High Temperature treatment; Cold: Cold treatment; ABA: Abscisic Acid treatment; NaCl: Sodium Chloride treatment; PEG: Polyethylene Glycol treatment; CV: Coefficient of Variance; *r*^2^: Coefficient of Determination.

**Table 4:** Expression stability values and ranking of HKGs in Chinese fir cotyledon as calculated by BestKeeper.

Rank Position	HT-Cotyledon		Cold-Cotyledon		PEG-Cotyledon		ABA-Cotyledon		NaCl-Cotyledon		Total-Cotyledon	
	CV	*r*^2^	CV	*r*^2^	CV	*r*^2^	CV	*r*^2^	CV	*r*^2^	CV	*r*^2^
1	*GAPDH*	*GAPDH*	*GAPDH*	*Actin*	*18S*	*EF1a*	*18S*	*18S*	*18S*	*EF1a*	*18S*	*Actin*
2	*18S*	*EF1a*	*UBQ*	*GAPDH*	*EF1a*	*18S*	*UBQ*	*Actin*	*GAPDH*	*UBQ*	*Actin*	*18S*
3	*EF1a*	*UBQ*	*Actin*	*18S*	*GAPDH*	*UBQ*	*Actin*	*UBQ*	*UBQ*	*Actin*	*UBQ*	*EF1a*
4	*UBQ*	*18S*	*18S*	*UBQ*	*UBQ*	*Actin*	*EF1a*	*GAPDH*	*Actin*	*GAPDH*	*GAPDH*	*UBQ*
5	*Actin*	*Actin*	*EF1a*	*EF1a*	*Actin*	*GAPDH*	*GAPDH*	*EF1a*	*EF1a*	*18S*	*EF1a*	*GAPDH*

Abbreviation: HT: High Temperature treatment; Cold: Cold treatment; ABA: Abscisic Acid treatment; NaCl: Sodium Chloride treatment; PEG: Polyethylene Glycol treatment; CV: Coefficient of Variance; *r*^2^: Coefficient of Determination.

**Table 5:** Best combination of HKGs based on the geNorm and RankAggreg programs.

Different Tissue	Treatment					
	Cold	HT	ABA	NaCl	PEG	Total
Cotyledon	*GAPDH*	*GAPDH*	*EF1a*	*18S*	*GAPDH*	*GAPDH*
	*UBQ*	*UBQ*	*UBQ*	*GAPDH*	*EF1a*	*UBQ*
Root	*Actin*	*GAPDH*	*GAPDH*	*Actin*	*EF1a*	*Actin*
	*EF1a*	*18S*	*UBQ*	*18S*	*Actin*	*EF1a*

Abbreviations: HT: High Temperature treatment; Cold: Cold treatment; ABA: Abscisic Acid treatment; NaCl: Sodium Chloride treatment; PEG: Polyethylene Glycol treatment.

## Supplementary Materials

Supplementary materials can be found at http://www.mdpi.com/1422-0067/17/8/1198/s1.
