# Supplementary material for: Screening and Validation of Housekeeping Genes of the Root and Cotyledon of Cunninghamia lanceolata under Abiotic Stresses by Using Quantitative Real-Time PCR
Source: Int J Mol Sci. 2016 Jul 28;17(8):1198. doi: 10.3390/ijms17081198 (PMC5000596; doi:10.3390/ijms17081198)
Supplement: Supplementary file 1 [file ijms-17-01198-s001.pdf]

Supplementary Materials: Screening and Validation of Housekeeping Genes of the Root and Cotyledon of *Cunninghamia lanceolata* under Abiotic Stresses by Using Quantitative Real-Time PCR

Wenlong Bao, Yanli Qu, Xiaoyi Shan and Yinglang Wan

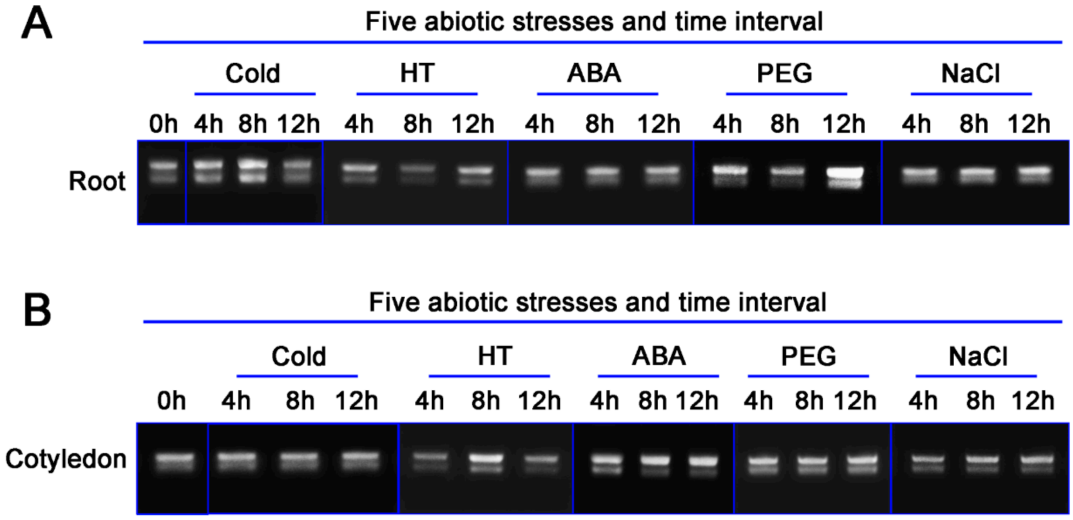

Figure S1. RNA gel electrophoresis of root (**A**) and cotyledon (**B**) sample sets.

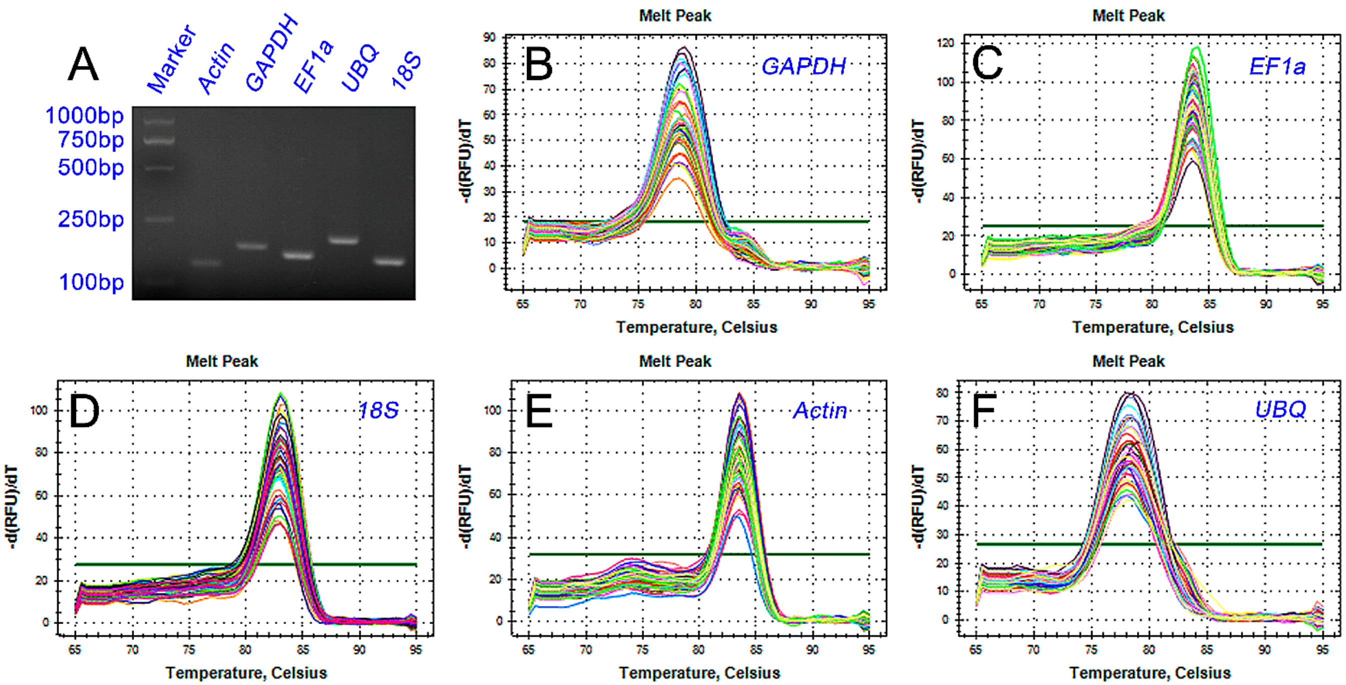

Figure S2. qRT-PCR amplification specificity and melt curve. Agarose gel electrophoresis of each HKG candidate gene (**A**); Melt curve of *GAPDH* (**B**); *EF1a* (**C**); *18S* (**D**); *Actin* (**E**) and *UBQ* (**F**). Gene fragments generated from qRT-PCR were isolated by 2.5% agarose gel electrophoresis. HT: High Temperature treatment; Cold: Cold treatment; ABA: Abscisic Acid treatment; NaCl: Sodium Chloride treatment; PEG: Polyethylene Glycol treatment.

Table S1. The transcriptome ID, *Arabidopsis* orthologous locus, primer sequences, amplicons length and PCR efficiencies for five candidate HKGs in Chinese fir.

Gene Abbreviation	Chinese Fir Transcriptome ID	Arabidopsis Orthologlocus	Primer Sequences (Forward/Reverse)	Amplicon Length (bp)	PCR Efficiency
Actin	comp117588-c0	AT3G18780	AAGCTCTCCTTTGTTGCTGTT/GACTTCTGGGCATCTGAATCT	138	2.05
GAPDH	comp51461_c0	AT1G79530	GCACCTATGTTTGTGGTTGGAGTA/ACCGTCTTCTGTGTAGCTGTTGTT	182	2.08
EF1α	comp126172_c0	AT5G60390	TGGCAAGGAGCTTGAGAAAGAACCCA/ACCCCAACAGCAACAGTCTGACGCAT	162	1.92
UBQ	comp129739_c0	AT1G65350.1	AATAAATGCTTCAAATGTCAGGCTA/TGAGATGGTCTGGTGATGTCGTGG	207	2.03
18S	comp134344_c0	AT3G57000	GGGAACATTATCACGGACAGCATCAAC/TGCGACTAATGGTCCAGATAGACTCCT	154	2.01

Table S2. Specific data of CV and r^2 (root).

HT-Root				Cold-Root				PEG-Root			
**Gene**	**CV**	**Gene**	**r^2**	**Gene**	**CV**	**Gene**	**r^2**	**Gene**	**CV**	**Gene**	**r^2**
UBQ	1.601	*EF1a*	0.694	*Actin*	0.271	*GAPDH*	0.13	*Actin*	0.995	*UBQ*	0.033
18s	1.618	*Actin*	0.884	*18s*	1.31	*18s*	0.144	*UBQ*	1.086	*GAPDH*	0.094
Actin	2.315	*UBQ*	0.992	*UBQ*	4.071	*UBQ*	0.326	*GAPDH*	2.344	*18s*	0.281
EF1a	5.188	*18s*	0.996	*GAPDH*	5.168	*Actin*	0.721	*18s*	2.75	*Actin*	0.399
GAPDH	14.128	*GAPDH*	0.98	*EF1a*	9.494	*EF1a*	1	*EF1a*	3.427	*EF1a*	0.764
**ABA-Root**				**NaCl-Root**				**Total-Root**			
**Gene**	**CV**	**Gene**	**r^2**	**Gene**	**CV**	**Gene**	**r^2**	**Gene**	**CV**	**Gene**	**r^2**
GAPDH	0.684	*Actin*	0.293	*Actin*	0.098	*GAPDH*	0.009	*Actin*	1.593	*UBQ*	0.002
Actin	0.747	*GAPDH*	0.719	*18s*	1.375	*EF1a*	0.162	*18s*	2.384	*18s*	0.189
UBQ	1.537	*18s*	0.792	*EF1a*	3.426	*Actin*	0.274	*UBQ*	2.717	*GAPDH*	0.359
18s	2.362	*UBQ*	0.863	*UBQ*	3.889	*18s*	0.848	*EF1a*	5.847	*Actin*	0.387
EF1a	2.767	*EF1a*	0.931	*GAPDH*	4.083	*UBQ*	0.922	*GAPDH*	6.208	*EF1a*	0.425

Abbreviation: HT: High Temperature treatment; Cold: Cold treatment; ABA: Abscisic Acid treatment; NaCl: Sodium Chloride treatment; PEG: Polyethylene Glycol treatment; CV: Coefficient of Variance; r^2: Coefficient of Determination.

Table S3. Specific data of CV and r^2 (cotyledon).

HT-Cotyledon				Cold-Cotyledon				PEG-Cotyledon			
**Gene**	**CV**	**Gene**	**r^2**	**Gene**	**CV**	**Gene**	**r^2**	**Gene**	**CV**	**Gene**	**r^2**
GAPDH	0.193	*GAPDH*	0.731	*GAPDH*	0.569	*Actin*	0.001	*18S*	1.273	*EF1a*	0.388
18S	0.244	*EF1a*	0.824	*UBQ*	0.674	*GAPDH*	0.026	*EF1a*	1.759	*18S*	0.545
EF1a	1.013	*UBQ*	0.867	*Actin*	1.617	*18S*	0.408	*GAPDH*	2.022	*UBQ*	0.638
UBQ	1.692	*18S*	0.966	*18S*	2.419	*UBQ*	0.726	*UBQ*	2.941	*Actin*	0.79
Actin	2.484	*Actin*	0.984	*EF1a*	2.874	*EF1a*	2.874	*Actin*	3.917	*GAPDH*	0.968
**ABA-Cotyledon**				**NaCl-Cotyledon**				**Total-Cotyledon**			
**Gene**	**CV**	**Gene**	**r^2**	**Gene**	**CV**	**Gene**	**r^2**	**Gene**	**CV**	**Gene**	**r^2**
18S	2.059	*18S*	0.806	*18S*	1.061	*EF1a*	0.529	*18S*	2.42	*Actin*	0.289
UBQ	2.343	*Actin*	0.824	*GAPDH*	2.908	*UBQ*	0.551	*Actin*	3.266	*18S*	0.419
Actin	2.502	*UBQ*	0.874	*UBQ*	4.024	*Actin*	0.78	*UBQ*	3.377	*EF1A*	0.692
EF1a	3.143	*GAPDH*	0.901	*Actin*	4.131	*GAPDH*	0.949	*GAPDH*	3.533	*UBQ*	0.767
GAPDH	3.651	*EF1a*	0.962	*EF1a*	5.384	*18S*	1	*EF1a*	3.887	*GAPDH*	0.794

Abbreviation: HT: High Temperature treatment; Cold: Cold treatment; ABA: Abscisic Acid treatment; NaCl: Sodium Chloride treatment; PEG: Polyethylene Glycol treatment; CV: Coefficient of Variance; r^2: Coefficient of Determination.

Table S4. Summary of results generated from different algorithms (root).

Different Algorithms	Tissue Under the Different Treatments					
	**HT-Root**	**Cold-Root**	**PEG-Root**	**ABA-Root**	**NaCl-Root**	**Total-Root**
NormFinder (Stab.)	*Actin*	*Actin*	*UBQ*	*GAPDH*	*Actin*	*Actin*
BestKeeper (CV)	*UBQ*	*Actin*	*Actin*	*GAPDH*	*Actin*	*Actin*
BestKeeper (r^2)	*GAPDH*	*EF1a*	*EF1a*	*EF1a*	*UBQ*	*EF1a*
geNorm (M value)	*18S*	*18S*	*Actin*	*Actin*	*18S*	*18S*
RankAggreg	*GAPDH*	*Actin*	*EF1a*	*GAPDH*	*Actin*	*Actin*

Abbreviation: HT: High Temperature treatment; Cold: Cold treatment; ABA: Abscisic Acid treatment; NaCl: Sodium Chloride treatment; PEG: Polyethylene Glycol treatment; Stab. : Stability; CV: Coefficient of Variance; r^2: Coefficient of Determination.

Table S5. Summary of results generated from different algorithms (cotyledon).

Different Algorithms	Tissue Under the Different Treatments					
	**HT-Cotyledon**	**Cold-Cotyledon**	**PEG-Cotyledon**	**ABA-Cotyledon**	**NaCl-Cotyledon**	**Total-Cotyledon**
NormFinder (Stab.)	*EF1a*	*UBQ*	*GAPDH*	*EF1a*	*GAPDH*	*GAPDH*
BestKeeper (CV)	*GAPDH*	*GAPDH*	*18S*	*18S*	*18S*	*18S*
BestKeeper (r^2)	*Actin*	*EF1a*	*GAPDH*	*EF1a*	*18S*	*GAPDH*
geNorm (*M* value)	*18S*	*Actin*	*18S*	*18S*	*18S*	*UBQ*
RankAggreg	*GAPDH*	*GAPDH*	*GAPDH*	*EF1a*	*18S*	*GAPDH*

Abbreviation: HT: High Temperature treatment; Cold: Cold treatment; ABA: Abscisic Acid treatment; NaCl: Sodium Chloride treatment; PEG: Polyethylene Glycol treatment; Stab. : Stability; CV: Coefficient of Variance; r^2: Coefficient of Determination.
